# The dual-IR sequence improves the inter-observer correlation in post-ablation atrial scar size measurements compared with the traditional IR sequence

**DOI:** 10.1186/1532-429X-15-S1-P72

**Published:** 2013-01-30

**Authors:** Sarah A Peel, Aruna Arujuna, James Harrison, Zhong Chen, Kawal Rhode, Jaswinder S Gill, Reza Razavi, Tobias Schaeffter, Rene M Botnar

**Affiliations:** 1 Division of Imaging Sciences and Bioengineering, King's College London, London, UK; 2 Department of Cardiology, Guy's and St. Thomas' NHS Foundation Trust, London, UK

## Background

A good correlation has been found between the extent of late gadolinium enhancement (LGE) and clinical outcome in patients who have undergone radiofrequency (RF) ablation for atrial fibrillation (AF). However, there can be strong residual blood signal in the traditional inversion-recovery (IR) sequence, which hampers scar visualization and causes poor repeatability of scar size measurements. The dual-IR sequence has previously been shown to improve blood suppression in LGE images of ventricular scar. We aimed to assess whether the superior blood suppression in the dual-IR pre-pulse improved the inter-observer variability of left atrial scar measurements compared with the IR sequence.

## Methods

The dual-IR pre-pulse consists of two non-selective inversion pre-pulses separated by two time delays TI1 and TI2. The TI1 and TI2 delays were optimized to achieve signal suppression in the T1-range 250-1400ms. Whereas the IR sequence can only null one T1 species (e.g. normal myocardium), the dual-IR pre-pulse simultaneously suppresses both the blood and normal myocardium whilst maintaining high signal in the scar.

11 patients (10 male, age 57±10yrs) underwent MR imaging using a 1.5T MR scanner (Philips Healthcare, the Netherlands) approximately 3 months after RF ablation for AF. Dual-IR imaging was performed at 20 minutes and compared to standard IR imaging at 25 minutes after 0.2 mmol/kg of Magnevist (Bayer Schering, Berlin) was administered. For each 3D image set, two blinded, independent, experienced readers used ITK-SNAP software to manually segment areas of LGE around the left atrial wall.

## Results

**Figure 1:**
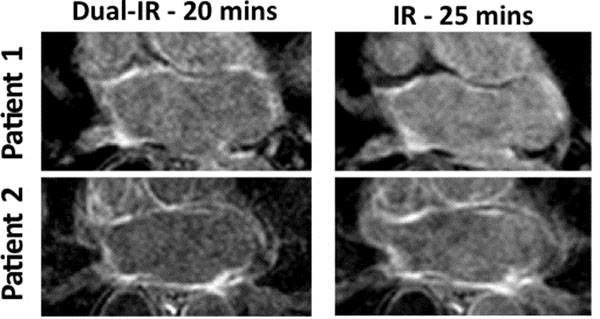
LGE atrial scar images using the dual-IR sequence and the IR sequence in two patients.

**Figure 2:**
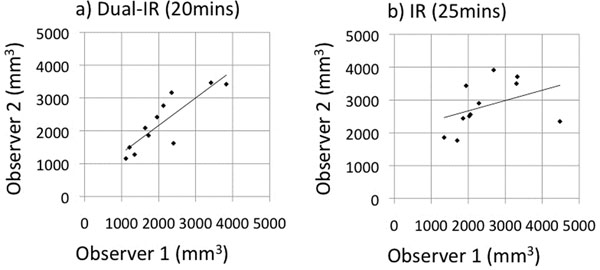
Correlation plots for total scar measurements (mm^3^) for the dual-IR sequence (a) and the IR sequence (b).

Dual-IR images achieved superior blood suppression at an earlier time point compared with IR images (Figure 1). There was no statistically significant difference between total scar size measurements on IR images and dual-IR images for either observer. The Pearson's correlation coefficient (R) for total scar measurements in the dual-IR images was two-fold higher than that in the IR images (Figure 2). R = 0.86 for dual-IR images compared with R = 0.39 for IR images.

## Conclusions

The dual-IR technique improves blood signal suppression and definition of the edges and boundaries of LGE areas. This leads to improved inter-observer variability in scar size quantification. As imaging can be performed earlier, it also has the potential to reduce the overall scan time.

## Funding

This work was funded by the British Heart Foundation award RE/08/003. The authors also acknowledge financial support from the Department of Health via the National Institute for Health Research (NIHR) comprehensive Biomedical Research Centre award to Guy's & St Thomas' NHS Foundation Trust in partnership with King's College London and King's College Hospital NHS Foundation Trust and the Centre of Excellence in Medical Engineering funded by the Wellcome Trust and EPSRC under grant number WT 088641/Z/09/Z.

